# Altered microRNAs related to synaptic function as potential plasma biomarkers for Alzheimer’s disease

**DOI:** 10.1186/s13195-019-0501-4

**Published:** 2019-05-15

**Authors:** Dolores Siedlecki-Wullich, Judit Català-Solsona, Cristina Fábregas, Isabel Hernández, Jordi Clarimon, Alberto Lleó, Merce Boada, Carlos A. Saura, José Rodríguez-Álvarez, Alfredo J. Miñano-Molina

**Affiliations:** 1 Institut de Neurociències and Dpt. Bioquímica i Biología Molecular, Universitat Autònoma de Barcelona, 08193 Cerdanyola del Vallès, Spain; 2 Centro de Investigación Biomédica en Red sobre Enfermedades Neurodegenerativas (CIBERNED), Madrid, Spain; 3 Research Center and Memory Clinic, Fundació ACE, Institut Català de Neurociències Aplicades, Universitat Internacional de Catalunya, Barcelona, Spain; 4 Memory Unit, Department of Neurology, Institut d’Investigacions Biomèdiques Sant Pau - Hospital de Sant Pau, Universitat Autònoma de Barcelona, Barcelona, Spain; 5 Dominick P. Purpura Department of Neuroscience, Albert Einstein College of Medicine, New York, NY 10461 USA

**Keywords:** Alzheimer’s disease, Mild cognitive impairment, Synapses, miRNAs, Plasma, Human, Biomarker, Frontotemporal dementia

## Abstract

**Background:**

Several evidences suggest that failure of synaptic function occurs at preclinical stages of Alzheimer’s disease (AD) preceding neuronal loss and the classical AD pathological hallmarks. Nowadays, there is an urgent need to identify reliable biomarkers that could be obtained with non-invasive methods to improve AD diagnosis at early stages. Here, we have examined plasma levels of a group of miRNAs related to synaptic proteins in a cohort composed of cognitive healthy controls (HC), mild cognitive impairment (MCI) and AD subjects.

**Methods:**

Plasma and brain levels of miRNAs were analysed in two different cohorts including 38 HC, 26 MCI, 56 AD dementia patients and 27 frontotemporal dementia (FTD) patients. D’Agostino and Pearson and Shapiro-Wilk tests were used to evaluate data normality. miRNA levels between groups were compared using a two-sided nonparametric Mann-Whitney test and sensitivity and specificity was determined by receiver operating characteristic curve analysis.

**Results:**

Significant upregulation of miR-92a-3p, miR-181c-5p and miR-210-3p was found in the plasma of both MCI and AD subjects. MCI patients that progress to AD showed higher plasma levels of these miRNAs. By contrast, no changes in miR-92a-3p, miR-181c-5p or miR-210-3p levels were observed in plasma obtained from a cohort of FTD.

**Conclusion:**

Our study shows that plasma miR-92a-3p, miR-181c-5p and miR-210-3p constitute a specific molecular signature potentially useful as a potential biomarker for AD.

**Electronic supplementary material:**

The online version of this article (10.1186/s13195-019-0501-4) contains supplementary material, which is available to authorized users.

## Background

It is estimated that more than 40 million people worldwide are affected by Alzheimer’s disease (AD) and it is expected that about 100 million could be affected by 2050. Unfortunately, there is no treatment to prevent or even reverse AD, making the finding of new therapeutic breakthroughs a huge challenge for our societies [1]. During the last decade, much effort has been devoted to the establishment of early AD biomarkers that could help to implement appropriate personalised care programmes before the onset of neurodegeneration and dementia. Moreover, effective early AD biomarkers could improve the design of clinical trials and help to reverse the continuous failures in the search of disease-modifying agents for AD obtained in the last years [2–4].

The best AD biomarkers achieved to date are based on neuroimaging methods (Aβ in the brain or hippocampal atrophy) or by the detection in the cerebrospinal fluid (CSF) of total tau, phospho-tau and Aβ_42_ [5–8]. Recently, a specific roadmap has been suggested to validate these neuroimaging- and CSF-derived biomarkers [9]. Unfortunately, all these potential biomarkers could not be used in routine clinical screening due to their invasiveness and economic limitations.

On the other hand, several evidences indicate that cognitive impairment observed in early stages of AD could be explained by alterations in synaptic function that precedes neurodegeneration [10]. Furthermore, some reports have shown that deregulation of synaptic proteins could be related to early cognitive dysfunction in experimental models of AD [11–14]. Thus, changes in the regulatory mechanisms involved in the expression of synaptic proteins could be valuable for assessing prognosis and the rate of cognitive decline in AD.

MicroRNAs (miRNAs) are small non-coding RNAs that play a significant role in local control of mRNA translation. In fact, a single miRNA could regulate the local expression of multiple proteins. Several studies have shown that some miRNAs control the formation, maturation and function of synapses [15] and alteration in their levels could underlie synaptic dysfunction in pathological states [16]. Notably, a number of specific miRNAs are misregulated in AD, including miRNAs implicated in the regulation of key genes involved in AD, such as APP or BACE1, or neuronal function such as glutamate receptors [17–20]. Extensive interest has been focused on identifying changes in specific miRNAs that could be used as AD biomarkers [21–25]. miRNAs can be found in the blood where they are transported in different structures that protect them from degradation [26, 27]. Since blood collection is an easy and non-invasive procedure, the identification of blood-based miRNA biomarkers for AD has drawn attention during the last years [28]. Altered levels of certain miRNAs in plasma of AD patients have been previously reported [21, 25, 29–32]; however, few data exist about changes in plasma levels of miRNAs that are related to synaptic protein function in AD [33]. In the present study, we have analysed several miRNAs related to synaptic proteins in plasma samples from mild cognitive impairment (MCI) and AD subjects. We have also monitored plasma samples from frontotemporal dementia (FTD) patients to verify the specificity of the obtained results. Our data suggest that miR-92a-3p, miR-181c-5p and miR-2 10-3p are potential and specific plasma biomarkers for AD.

## Methods

### Subjects

Plasma samples analysed in this study were obtained from two different cohorts. The first cohort provided by Fundació ACE (Barcelona, Spain) includes 14 HC, 26 MCI and 56 AD dementia patients. The second cohort was recruited at the Memory Unit of the Hospital Sant Pau (Barcelona, Spain) and consists of 24 HC and 27 FTD patients. Participants were clinically diagnosed by neurologists and classified according to internationally accepted diagnostic criteria [34]. Specifically, MCI subjects fulfilled Petersen’s diagnostic criteria [35], and their neuropsychological assessment was done by the previously validated NBACE battery tests [36]. FTD participants include 19 patients with possible or probable behavioural variant [37] and 6 with semantic variant of primary progressive aphasia [38]. Two patients with FTD were additionally diagnosed with concomitant ALS according to El Escorial criteria [39]. Demographic and clinical characteristics of cohorts 1 and 2 are summarised in Table 1. Brain tissue samples were provided by 3 different Spanish centres: Fundación Cien, Hospital Clinic-IDIBAPS and Hospital Universitario Fundación Alcorcón. Demographic and clinical characteristics of the subjects are listed in Additional file 1: Table S1. Brain tissue from entorhinal cortex (*n* = 13), hippocampus (*n* = 49) and cerebellum (*n* = 30) was analysed.

**Table 1:** The expression levels of miRNAs related to synaptic proteins

Cohort 1				Cohort 2	
	HC	MCI	AD	HC	FTD
Cohort size	14	26	56	24	27
Men/women	7/7	10/16	15/41	11/13	17/10
Age (years)	68.29 ± 8.99	72.0 ± 8.49	77.77 ± 6.69	67.03 ± 5.05	68.87 ± 7.48
GDS	2.07 ± 0.26	3.15 ± 0.37	4.64 ± 1.02	1 ± 0	3.76 ± 0.99
MMSE	29.21 ± 1.05	26.92 ± 2.22	16.05 ± 7.23	28.5 ± 1.69	25.5 ± 3.76

All data are shown as mean ± SD

*HC* cognitively healthy controls, *MCI* mild cognitive impairment, *AD* Alzheimer’s disease, *FTD* frontotemporal dementia, *MMSE* Mini-Mental State Examination, *GDS* Global Deterioration Scale

### Sample processing, RNA extraction and reverse transcription

Blood samples were collected in EDTA-containing tubes, as recommended [40]. After 20 min centrifugation (2500×*g*), plasma was separated, aliquoted and stored at − 80 °C until use. Plasma samples were thawed on ice for RNA extraction, and hemolysis of each sample was analysed at the time measuring absorbance at 414/375 nm (414/375 ratio > 1.4 were considered hemolysed) [41]. RNA was isolated from 200 μL of plasma, using the miRNeasy RNA isolation kit (Qiagen) following the manufacturer’s indications. Two microlitres of RNA was reverse-transcribed to cDNA using TaqMan™ Advanced miRNA cDNA Synthesis Kit (Thermo Fisher Scientific).

Postmortem brains were quickly homogenated in dry ice to prevent thawing, and RNA was isolated from 20 to 50 mg of tissue from each brain area using *miR*Vana miRNA Isolation Kit (Thermo Fisher Scientific) following the manufacturer’s instructions. RNA quality was evaluated using the Agilent 2100 bioanalyzer. Samples with RNA integrity number (RIN) under 4 were excluded [40, 42]. Ten nanogrammes of RNA were reverse-transcribed to cDNA using TaqMan™ MicroRNA Reverse Transcription Kit (Thermo Fisher Scientific).

### miRNAs quantification by RT-qPCR

Quantitative real-time PCR (RT-qPCR) was performed from 5 μL of 1/10 diluted cDNA (plasma samples) using TaqMan Fast Advanced Master Mix and TaqMan Advanced miRNA Assays (Thermo Fisher Scientific) or from 1.33 μL of cDNA (tissue samples) using TaqMan™ Universal Master Mix II, with UNG. Applied Biosystems 7500 Fast instrument was used for amplification. Samples were run in duplicate, and internal control samples were repeated in every plate to avoid batch effects. Raw Ct data acquired using the 7500 Software v2.0.6 (Applied Biosystems) was exported to LinRegPCR software to calculate the amplification efficiency for each reaction. Reactions with amplification efficiency below 1.6 were discarded. Ct values and average efficiencies obtained from LinRegPCR were used to analyse miRNA levels by the comparative ΔΔCt method [43]. To date, there is no consensus on the use of particular reference genes for miRNA level normalisation in AD studies. Therefore, in this study, the stability of some described reference genes was evaluated using the NormFinder algorithm [44]. hsa-miR-191-5p and hsa-miR-484 were identified as the most stable reference genes along with all plasma samples. In addition, hsa-miR-191-5p and hsa-miR-484 showed higher correlation than other candidates (Spearman’s correlation coefficient *r* = 0.89; *P* < 0.0001). U18 and RNU48 were selected for tissue data normalisation. miRNA levels were normalised versus the geometric mean of selected reference genes, to compensate abundance differences between miRNAs and prevent statistical outliers [45].

### Western blotting

Human brain tissue was lysed in ice with cold RIPA buffer (50 mM Tris base pH 7.4, 150 mM NaCl, 2 mM EDTA, 1% NP40, 0.5% Triton X-100, 0.1% SDS, 1 mM Na_3_VO_4_, 25 mM NaF, 1 mM PMSF, 1/100 protease inhibitors and 1/100 phosphatases inhibitors cocktail), sonicated and centrifuged. The supernatant was recovered, and concentration was determined by BCA assay. An equal amount of protein was loaded in 10–12% polyacrylamide gels and separated by electrophoresis under denaturing conditions (SDS-PAGE). Proteins were then transferred to nitrocellulose membrane (GE Healthcare), and membranes were further incubated with blocking solution (10% dry milk, 0.1% BSA, pH 7.4) for 1 h and washed with phosphate buffer saline-Tween (PBT-T). Next, membranes were incubated with primary antibodies (anti-NPTX1 (1:1000, BD), anti-NPTXR (1:500, Santa Cruz) and anti-b-actin (1:5000, Sigma) overnight at 4 °C. After washing membranes with PBT-T, they were incubated at room temperature for 1 h with secondary peroxidase-coupled antibodies (mouse or rabbit as needed) prepared in blocking solution. After repeated washes, proteins were detected by chemoluminescence reaction using ECL Western Blotting reagent (GE Healthcare). Immunoblots were analysed by densitometry using ImageJ (National Institutes of Health, Bethesda, MD), and protein levels were corrected for corresponding loading control.

### Statistical analysis

Ct values were normalised versus the Ct mean of the control group and log2 transformed. D’Agostino and Pearson and Shapiro-Wilk tests were used to evaluate data normality. miRNA levels from controls, and patients were compared using a two-sided nonparametric Mann-Whitney test. Bonferroni correction was applied for multiple comparisons when needed. Non-parametric Spearmen’s rank correlation test was used to determine the correlation between miRNA expression levels and age. *P* values < 0.05 were considered statistically significant. ROC curve analysis under a nonparametric approach was used to obtain the area under de curve (AUC) to evaluate sensitivity and specificity of each miRNA as a predictive biomarker. Logistic regression was applied to evaluate biomarker combination by ROC curve analysis. Statistical analysis was performed using GraphPad Prism software v6.01 (GraphPad Software Inc., CA, USA) and MedCalc (v17.9.7).

## Results

### Upregulation of miR-92a, miR-181c and miR-210 in plasma of MCI and AD patients

**Fig. 1:**
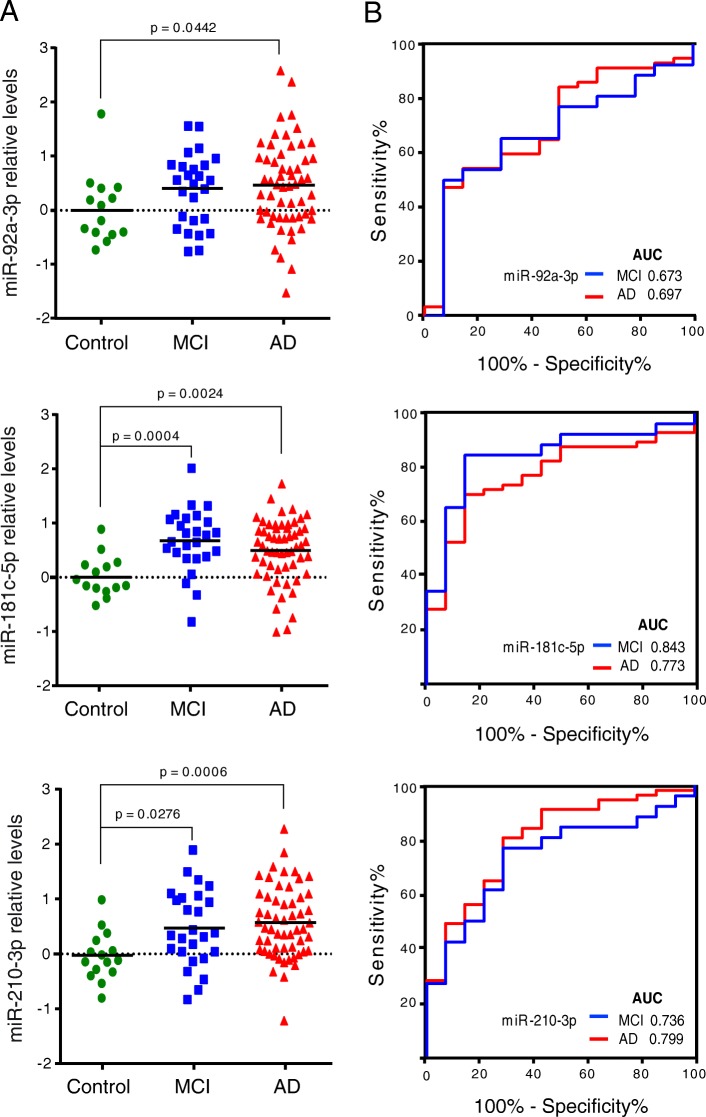
Circulating miRNA levels at different stages of AD pathology compared with cognitively normal controls (**a**). Log2 transformed data were normalised versus the geometric mean of miR-191-5p and miR-484 levels. Statistical significance was evaluated by the Mann-Whitney *U* test followed by the Bonferroni correction for multiple comparisons. *P* values < 0.05 were considered statistically significant. **b** Receiver operating characteristic (ROC) curve analysis was performed to distinguish MCI (blue) and AD (red) cases from healthy controls. The area under the curve (AUC) is shown for each miRNA and stage

The expression levels of miRNAs related to synaptic proteins, miR-92a-3p, miR-181c-5p, miR-210-3p and miR-584-5p (Additional file 1: Table S2), were analysed by RT-qPCR in the plasma of a cohort consisting of 14 HC, 26 MCI and 56 sporadic AD subjects (Table 1). We found a significant increase in the levels of miR-92a-3p, miR-181c-5p and miR-210-3p in plasma from AD patients when compared with HC (Fig. 1a; miR-92a-3p: *P* = 0.0442, log2 fold change = 0.52; miR-181c-5p: *P* = 0.0024, log2 fold change = 0.67; miR-210-3p: *P* = 0.0006, log2 fold change = 0.60). A significant increase was also observed in MCI plasma samples for miR-181c-5p (*P* = 0.0004, log2 fold change = 0.80) and miR-210-3p (*P* = 0.0276, log2 fold change = 0.49) whereas an increasing trend was observed for miR-92a-3p (*P* = 0.0798, log2 fold change = 0.55). By contrast, no apparent changes were observed in miR-584-5p expression levels between HC and MCI or AD subjects (data not shown). Statistical correlation analysis revealed that those changes were not due to the increasing age in the MCI and AD groups (Additional file 1: Figure S2). Also, no differences between sexes were observed in HC, MCI and AD groups (Additional file 1: Figure S3).

**Fig. 2:**
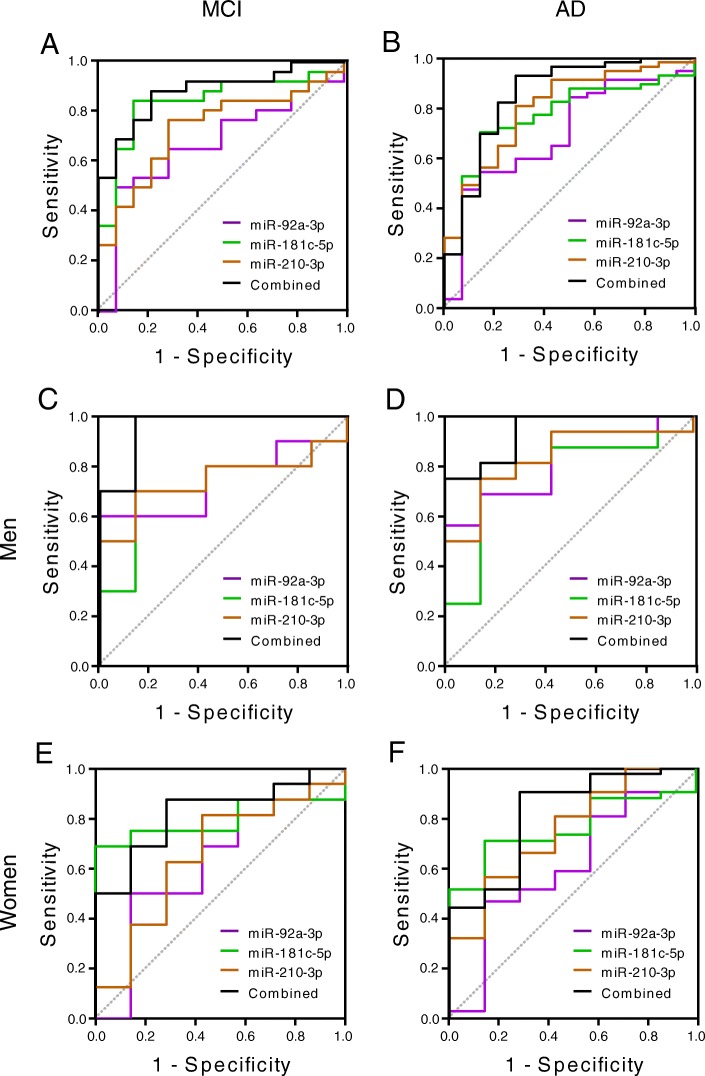
miRNAs were combined to reach the best predictive value. ROC curve analysis for MCI (**a**) and AD (**b**) cases are shown. Values for men (**c**, **d**) and women (**e**, **f**) are represented separately

Next, ROC curve analysis was performed to evaluate the diagnostic potential of miR-92a-3p, miR-181c-5p and miR-210-3p (Fig. 1b). The values obtained for miR-92a-3p, miR-181c-5p and miR-210-3p when HC were compared with MCI subjects were as follows: AUC values of 0.67, 0.84 and 0.74, respectively; 50.00%, 84.62% and 76.92% of sensitivity, respectively; and 92.86%, 85.71% and 71.43% of specificity, respectively (Table 2). AUC values for miR-92a-3p, miR-181c-5p and miR-210-3p when AD subjects were compared to HC were 0.70, 0.77 and 0.80, respectively. Whereas miR-92a-3p has 47.37% of sensitivity and 92.86% of specificity, miR-181c-5p has 70.18% of sensitivity and 85.71% of specificity and miR-210-3p has 80.70% of sensitivity and 71.43% of specificity (Table 3). ROC curve analysis using logistic regression for miR-92a-3p, miR-181c-5p and miR-210-3p combination yielded a better diagnostic value for MCI and AD, suggesting that these miRNAs together could be used as a molecular signature for early diagnosis of MCI and/or AD. We determined an AUC value of 0.893, a sensitivity of 84.62% and a specificity of 85.71% for distinguishing MCI from HC (Fig. 2a and Table 2). This molecular miRNA signature provided an AUC value of 0.855, a sensitivity of 92.86% and a specificity of 71.43% when AD samples were compared to HC (Fig. 2b and Table 3).

**Table 2:** Individual and signature miRNAs performance characteristics in predicting MCI stage. Signature miRNA performance characteristics in women and men

miRNA/signature	AUC	Sensitivity %	Specificity %	Youden Index J	*P* value
miR-92a-3p	0.673	50.00	92.86	0.428	0.0563
miR-181c-5p	0.843	84.62	85.71	0.703	< 0.0001
miR-210-3p	0.736	76.92	71.43	0.483	0.0038
miR-92a-3p/miR-210-3p	0.731	73.08	71.43	0.445	0.0050
miR-92a-3p/miR-181c-5p	0.838	84.62	78.57	0.632	< 0.0001
miR-181c-5p/miR-210-3p	0.865	88.46	78.57	0.670	< 0.0001
miR-92a-3p/miR-181c-5p/ miR-210-3p	0.893	84.62	85.71	0.703	< 0.0001
Women	0.821	87.50	71.43	0.589	0.0005
Men	0.957	100.0	85.71	0.857	< 0.0001

*AUC* area under the curve

**Table 3:** Individual and signature miRNAs performance characteristics in predicting AD. Signature miRNAs performance characteristics in women and men

miRNA/signature	AUC	Sensitivity %	Specificity %	Youden Index J	*P* value
miR-92a-3p	0.697	47.37	92.86	0.402	0.0124
miR-181c-5p	0.773	70.18	85.71	0.559	< 0.0001
miR-210-3p	0.799	80.70	71.43	0.521	< 0.0001
miR-92a-3p/miR-210-3p	0.807	87.72	64.29	0.520	< 0.0001
miR-92a-3p/miR-181c-5p	0.787	84.21	71.43	0.556	0.0001
miR-181c-5p/miR-210-3p	0.853	84.21	78.57	0.628	< 0.0001
miR-92a-3p/miR-181c-5p/ miR-210-3p	0.855	92.86	71.43	0.644	< 0.0001
Women	0.815	90.24	71.43	0.617	0.0009
Men	0.938	75.0	100.0	0.750	< 0.0001

*AUC* area under the curve

We also looked for eventual sex-dependent differences in the diagnostic potential of the examined miRNAs (Fig. 2). We found a slightly better diagnostic value for men than for women to distinguish MCI from HC subjects (Table 2): (a) the AUC values for women and men are 0.82 and 0.96 respectively; (b) 87.50% sensitivity for women and 100% for men; and (c) 71.43% and 85.71% specificity for women and men, respectively. On the other hand, the values obtained when comparing AD with HC subjects indicated a better diagnostic specificity and a lower sensitivity for men than for women (Table 3): (a) the AUC values for women and men are 0.82 and 0.94; (b) 90.24% and 75.00% sensitivity for women and men; and (c) 71.43% and 100% specificity for women and men, respectively.

**Fig. 3:**
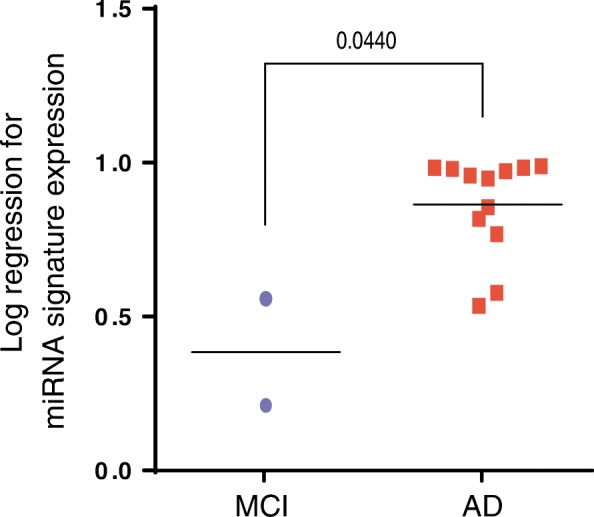
MCI patients were separated according to follow-up diagnosis. From 26 patients diagnosed as MCI in the first place, 2 remained stable and 12 have progressed to AD (see Additional file 1: Table S3). miRNA expression levels were combined using log regression approach. Mean of miRNA signature values for each group is shown. Statistical significance was evaluated by the Mann-Whitney *U* test. *P* values < 0.05 were considered statistically significant

In order to provide further support to the value of our molecular miRNA signature as a molecular biomarker for early AD diagnosis, we have followed up 19 of the 26 MCI subjects for 1 to 12 years (Additional file 1: Table S3). Three subjects diagnosed as MCI (11.5%; P4, P8 and P22) showed no cognitive impairment after 1 (P4 and P22) or 4 (P8) years, and another two (P14 and P18) were still diagnosed as MCI after 3 and 11 years. The rest of the cases, all developed dementia being AD the most prominent. Only one patient progressed to FTD (P7) and another to vascular dementia (P11; Additional file 1: Table S3). Interestingly, we have observed that all the MCI patients that progressed to AD have higher values of the miRNA signature compared to the MCI patients that did not evolve to AD (Fig. 3).

### Expression levels of miR-92a, miR-181c and miR-210 in AD brains

To investigate whether the observed changes in miR-92a-3p, miR-181c-5p and miR-210-3p levels were associated to changes in the brain, we measured their levels in the entorhinal cortex, hippocampus and cerebellum from cognitively normal controls (Braak I/II) and early (Braak III/IV) and late (Braak V/VI) AD patients (Additional file 1: Table S1 and Additional file 1: Figure S1). We found that miR-92a-3p, miR-181c-5p and miR-210-3p levels are slightly increased in the entorhinal cortex at early and late AD compared to HC. However, it only reached statistical significance in the case of miR-92a-3p when compared to late AD (Braak V-VI; Additional file 1: Figure S1A), probably due to the small number of samples analysed in this region. A similar trend was observed for miR-92a-3p and miR-181c-5p in the hippocampus (Additional file 1: Figure S1B). No significant changes of miR-210-3p in the hippocampus or in miR92a-3p, miR-181c-5p and miR-210-3p in the cerebellum were observed at any disease stage (Additional file 1: Figure S1B, C). Thus, although there is an overall trend to increase in the hippocampus and entorhinal cortex, it is not possible to conclude that miRNA changes observed in plasma are related to those observed in the brain since they lack global statistical significance. miR-181c-5p has neuronal pentraxin 1 (NPTX1) and neuronal pentraxin receptor (NPTXR) as potential targets. Since miR-181c-5p is increased in the entorhinal cortex, we next examined the protein levels of NPTX1 and NPTXR wanted to check whether this increase coincided with a change in the levels of these proteins. As shown in Additional file 1: Figure S3, we observed a decrease in NPTX1 and NPTXR although it did not reach statistical significance likely due to the low number of samples.

### Expression levels of miR92a, miR181c and miR210 are not affected in FTD patients

**Fig. 4:**
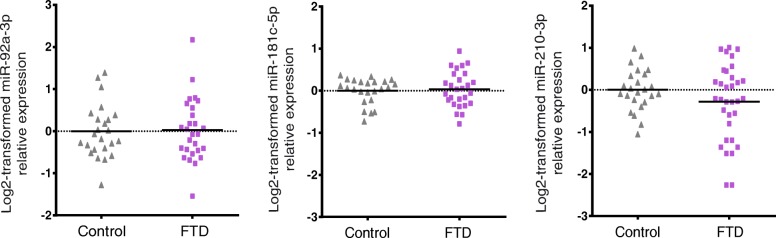
Plasma miRNA levels in FTD compared to cognitively normal controls. Log2 transformed data were normalised versus the geometric mean of miR-191-5p and miR-484 levels. Statistical significance was evaluated by the Mann-Whitney *U* test. *P* values < 0.05 were considered statistically significant

In order to determine whether the changes observed in miR-92a-3p, miR-181c-5p and miR-210-3p in plasma were specific of MCI and AD subjects, we decided to analyse plasma samples from a cohort of FTD patients. None of the abovementioned miRNAs were altered in FTD (Fig. 4) suggesting that the changes observed could be specific for MCI and AD subjects.

## Discussion

The search of blood-based biomarkers for early detection of AD has gained increasing attention during the last decade due to the non-invasiveness of the procedure and its low economical costs when compared to the use of CSF or neuroimaging techniques, which would potentially allow their use in routine tests worldwide. Among the diversity of molecules and factors that could be analysed in blood-derived samples, miRNAs represent one of the most promising approaches to identify peripheral AD biomarkers [7, 17, 20, 46] since they are relatively abundant and highly stable in blood.

Several reports have performed miRNAs profiling in brain and CSF that have identified numerous miRNAs as potential biomarkers to detect AD [46–49]. Whereas some studies have focused on miRNAs regulating specific AD-related proteins, little attention has been devoted to miRNAs involved in the regulation of synaptic proteins. In the present study, we have analysed plasma levels of specific miRNAs related to the regulation of synaptic proteins, especially glutamatergic synapses, in HC, MCI and AD patients. Thus, this work complements other reports that have suggested the potential of miRNAs as biomarkers for AD.

From the different miRNAs analysed, we have observed a significant increase in miR-92a-3p, miR-181c-5p and miR-210-3p plasma levels in MCI and AD. Moreover, an increase in the levels of these three miRNAs was observed in AD entorhinal cortex. However, only miR-92a-3p changes reached statistical significance probably due to the low number of available samples used. No significant changes in hippocampal levels were observed for miR-181c-5p and miR-210-3p. Hence, our data adds on many previous reports indicating that miRNA changes between brain and CSF/plasma samples do not necessarily run in parallel [22].

Among other targets, miR181c-5p and miR-210-3p have neuronal pentraxin 1 (NPTX1) and neuronal pentraxin receptor (NPTXR) as potential targets. Indeed, it has been described that miR-210-3p regulates the expression of NPTX1 and NPTXR [50]. These proteins are involved in the recruitment and clustering of AMPA receptors in postsynaptic terminals, organising and modelling the activity of glutamatergic synapses in the hippocampus affecting their synaptic plasticity [51, 52]. Thus, an increase of these miRNAs would reduce the levels of NPTX1 and NPTXR and could contribute to the glutamatergic synaptic dysfunction and early cognitive impairment present in AD prior to neurodegeneration. In fact, the increase in miR-181c-5p coincided with a decrease in NPTX1 and NPTXR protein in the entorhinal cortex.

Based on the observed increase in miR-92a-3p, miR-181c-5p and miR-210-3p, we propose that these miRNAs constitute a molecular signature that could be used as a biomarker for MCI and AD. The AUC values of this molecular signature are similar (0.893 accuracy, 84.6% sensitivity and 85.71% sensitivity) to the ones obtained in other studies that have proposed either a 12-miRNA signature that distinguishes MCI with 0.842 accuracy, 81.1% specificity and 87.7% sensitivity in blood [29] or a 9-miRNAs serum signature for AD cases identification with an AUC value of 0.978, 93.4% sensitivity and 98.8% of specificity [53]. Our results are the first that described an alteration of miR-92a-3p and miR-210-3p levels in biological fluids from AD patients. Only a previous report showed that soluble Aβ secreted from 7PA2 cells produced an increase in miR-210 levels in a mature primary culture of cortical neurons [54] and another study by Cogswell and co-workers reported that the levels of miR-92 were enhanced in AD hippocampus [47]. On the other hand, the observed increase in miR-181c-5p in plasma of MCI and AD subjects is challenged by previous reports reporting opposite observations. For instance, a decrease in miR-181c levels was reported in serum [20, 30, 53] and CSF [47] from AD patients. The causes of these discrepancies are currently unclear.

We were able to follow up for several years the evolution of the MCI patients included in our study, and we observed that the signature values in the plasma of the MCI patients that progressed to AD were significantly higher than the values found in the MCI patients that did not progress to dementia. Thus, plasma levels of miR-92a-3p, miR-181c-5p and miR-210-3p could not only be used as a biomarker signature for MCI and AD but might also indicate whether a patient with MCI will progress to AD. To our knowledge, this is the first study in which miRNAs levels in MCI patients are associated with the diagnosis of AD. However, we are aware that due to the low number of MCI patients that did not progress to AD, further studies with more samples should be done before we can conclude that plasma levels of miR-92a-3p, miR-181c-5p and miR-210-3p are a good prognostic signature for the progression from MCI to AD.

Several studies have shown that AD-associated changes in miRNAs expression are also found in other types of dementia such as frontotemporal dementia or dementia with Lewy bodies [55]. Thus, those miRNAs could not be considered as eventual specific biomarkers for AD. For example, some reports have shown that miR-132 levels are downregulated in AD [46, 49, 56] and similar results were obtained in other neurodegenerative diseases [19, 55, 57], suggesting that the alteration of this miRNA is linked to common pathological mechanisms in neurodegenerative diseases. In order to know whether this coincidence was also observed with miR-92a-3p, miR-181c-5p and miR-210-3p levels, we analysed their levels in plasma samples from a cohort of FTD patients. The results obtained indicate that no changes exist in our molecular miRNA signature between HC and FTD samples, suggesting that the increase in miR-92a-3p, miR-181c-5p and miR-210-3p levels could be specific for AD.

The existence of sex differences in AD is well established. Not only in the prevalence but also in the severity of the disease progression, being suggested that women could be more susceptible than men to the neuropathological cascade of AD [58]. Thus, it has become more necessary to introduce the effects of sex in the studies [59]. We have analysed whether differences exist between men and women and we did not find significant differences between sexes neither in the levels of the miRNAs nor in their value as a molecular signature. Only a slightly higher AUC was obtained in men to predict MCI (0.957 vs 0.821) or AD (0.938 vs 0.815) compared to women. Thus, data from the studied cohort supports that our miRNA signature would be equally effective as a biomarker for AD in both men and women.

In summary, this study provides data supporting that a plasma miRNA signature composed of miR-92a-3p, miR-181c-5p and miR-210-3p could be useful as a non-invasive clinical biomarker for diagnosis of AD that could be used for improving current clinical trials and in future therapeutic strategies. Moreover, the levels of these miRNAs in MCI patients could predict the progression to AD dementia. Further studies are needed to assess whether changes in this miRNA signature correlates with known AD biomarkers. Likewise, obtaining additional data from other MCI and AD cohorts would be necessary to fully validate the data presented in this study and to establish this miRNA signature as a reliable biomarker for early stages of AD.

## Conclusions

Deficits in synaptic function are likely to be involved in the development of AD in its preclinical stages. Here, we have analysed the levels of specific miRNAs related to synaptic proteins finding that the levels of miR-92a-3p, miR-181c-5p and miR-210-3p are deregulated in plasma from MCI and AD subjects while no changes are observed in plasma from FTD patients. Moreover, the analysis combining these three miRNAs yielded high diagnosis accuracy for distinguishing both MCI and AD subjects from healthy controls and could also predict MCI progress to AD. Thus, we propose plasma levels of miR-92a-3p, miR-181c-5p and miR-210-3p as a molecular signature to be used as a novel biomarker for AD.

## Additional file

Additional file 1: **Figure S1.** miRNA levels in human entorhinal cortex (A), hippocampus (B) and cerebellum (C) at different stages of AD pathology compared with cognitively healthy controls. **Figure S2.** Correlation plots for plasma miRNAs expression levels vs age. **Figure S3.** Circulating miRNAs levels comparison between sexes. **Figure S4.** NPTX1 and NPTXR protein levels in AD human entorhinal cortex. **Table S1.** Tissue samples information. **Table S2.** Validated miRNA-target interactions for candidate miRNAs based on miRWalk2.0 database. Only selected synaptic-related targets are shown. **Table S3.** Follow-up of MCI patients. (PDF 1295 kb)

## Abbreviations

AD: Alzheimer’s disease
AUC: Area under the curve
CSF: Cerebrospinal fluid
FTD: Frontotemporal dementia
HC: Healthy controls
MCI: Mild cognitive impairment
miRNAs: MicroRNAs
NPTX1: Neuronal pentraxin 1
NPTXR: Neuronal pentraxin receptor
ROC: Receiver operating characteristic
RT-qPCR: Quantitative real-time PCR
