# Supplementary material for: Altered microRNAs related to synaptic function as potential plasma biomarkers for Alzheimer’s disease
Source: Alzheimers Res Ther. 2019 May 15;11:46. doi: 10.1186/s13195-019-0501-4 (PMC6521366; doi:10.1186/s13195-019-0501-4)
Supplement: Supplementary file 1 — Figure S1. miRNA levels in human entorhinal cortex (A), hippocampus (B) and cerebellum (C) at different stages of AD pathology compared with cognitively healthy controls. Figure S2. Correlation plots for plasma miRNAs expression levels vs age. Figure S3. Circulating miRNAs levels comparison between sexes. Figure S4. NPTX1 and NPTXR protein levels in AD human entorhinal cortex. Table S1. Tissue samples information. Table S2. Validated miRNA-target interactions for candidate miRNAs based on miRWalk2.0 database. Only selected synaptic-related targets are shown. Table S3. Follow-up of MCI patients. (PDF 1295 kb) [file 13195_2019_501_MOESM1_ESM.pdf]

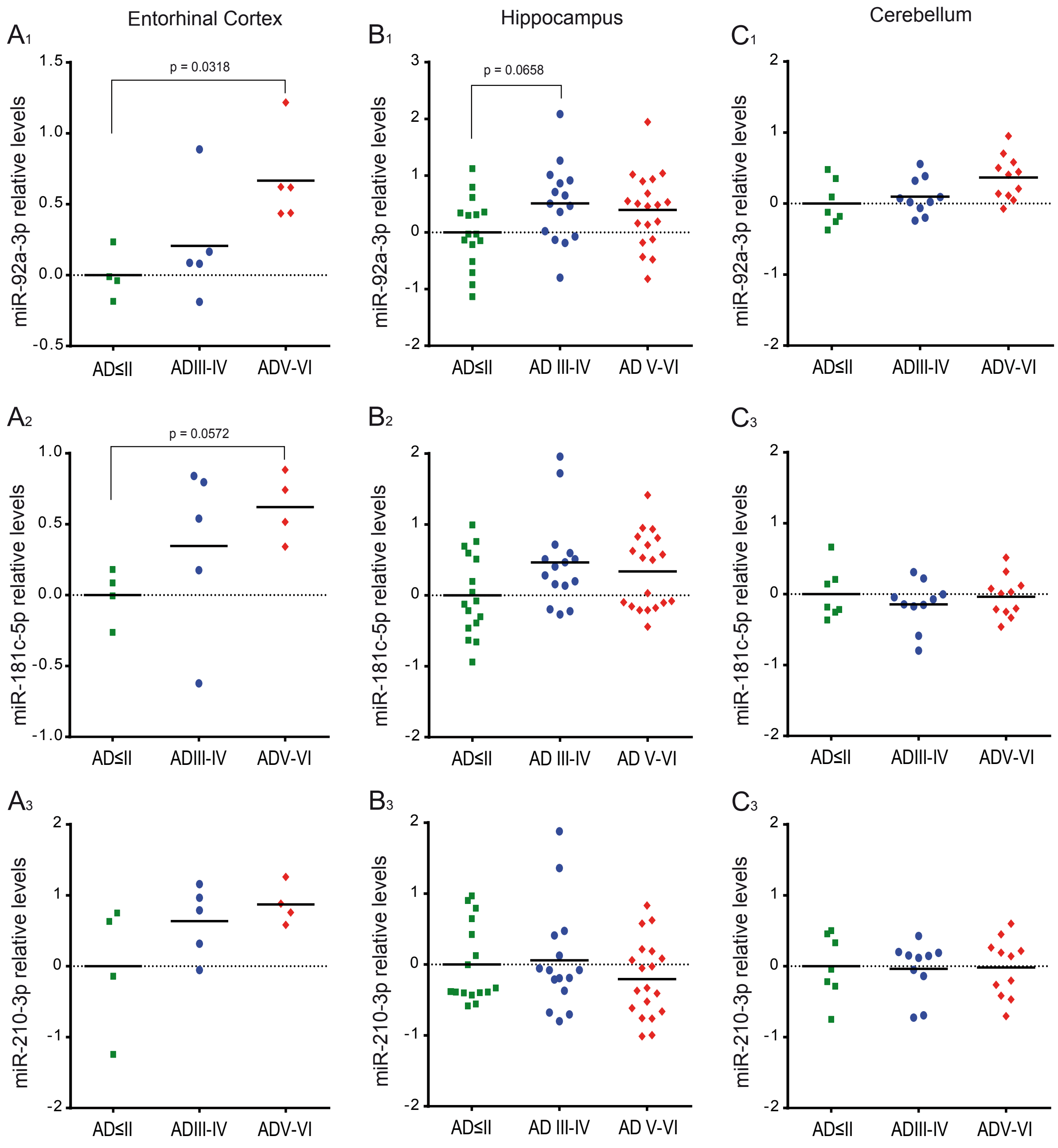

Figure S1. miRNA levels in human entorhinal cortex (A), hippocampus (B) and cerebellum (C) at different stages of AD pathology compared with cognitively healthy controls. Log2 transformed data were normalised versus the geometric mean of U18 and U48 levels. Statistical significance was evaluated by Mann-Whitney U test followed by Bonferroni correction for multiple comparisons.

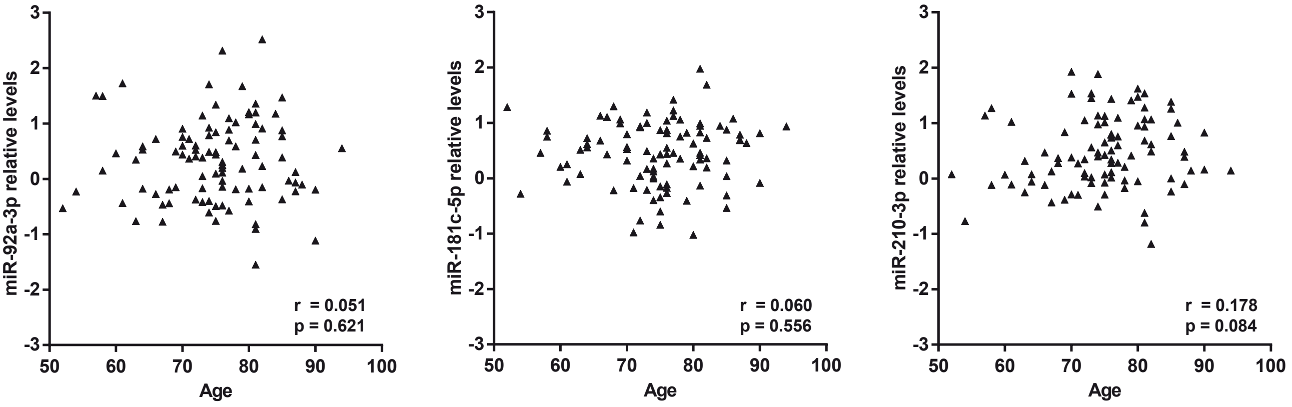

Figure S2. Correlation plots for plasma miRNAs expression levels vs age. Spearman correlation coefficients (r) and p-values are shown.

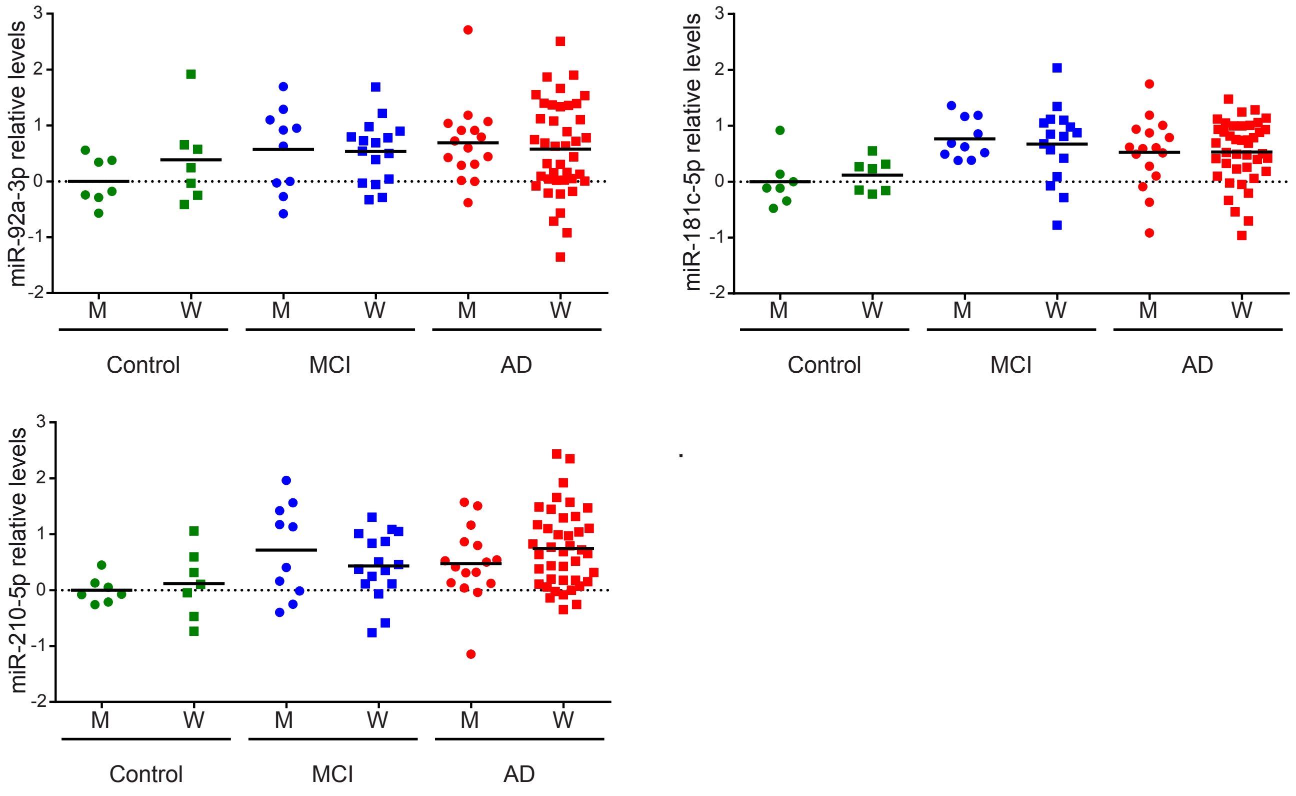

Figure S3. Circulating miRNAs levels comparison between sexes. Women (W) and men (M) levels were compared in each group. Log2 transformed data were normalised vs. the geometric mean of miR-191-5p and miR-484 levels. Data was evaluated by Mann-Whitney U test followed by Bonferroni correction for multiple comparisons. No statistically significant differences were observed between women and men.

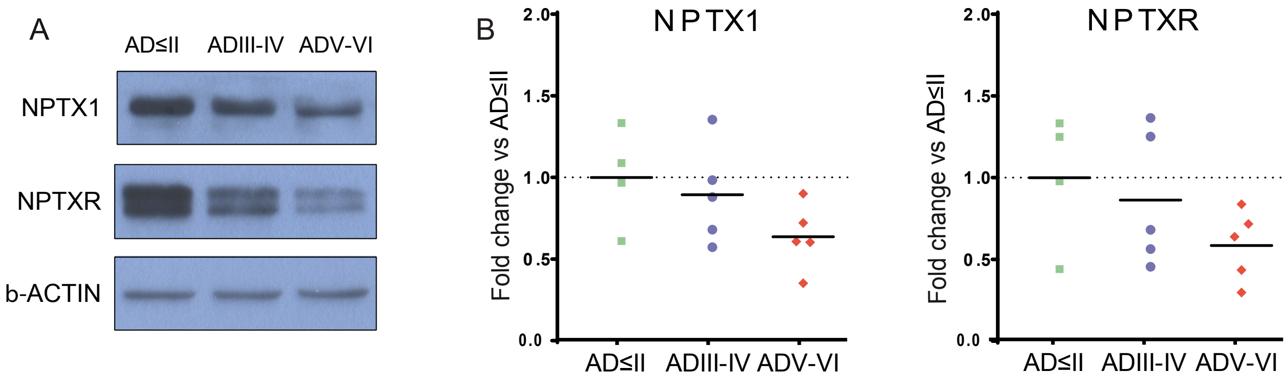

Figure S4. NPTX1 and NPTXR protein levels in AD human entorhinal cortex. Representative western blot (A) and densitometry values for all analysed samples (B) are shown.

Braak stage	Control<br>AD≤II	Early AD<br>ADIII-IV	AD<br>ADV-VI
	**Entorhinal cortex**		
Cohort size	4	5	4
M/F	3/1	3/1	0/4
Age (years)	81.7±5.5	88.2±6.7	83.5±3.1
PMD (h)	5.6±1.1	5±2.6	4.7±2.3
RIN	6.0±0.8	6.2±0.7	6.5±0.8
	**Hippocampus**		
Cohort size	19	12	18
M/F	10/9	8/4	6/12
Age (years)	72±13.2	85±5.9	81.2±5.9
PMD (h)	6.0±2.8	6.1±2.3	6.8±4.8
RIN	6.4±0.9	6.2±0.7	6.3±1
	**Cerebellum**		
Cohort size	8	11	11
M/F	3/5	8/3	1/10
Age (years)	78.63±12.69	85.36±6.05	82.36±5.16
PMD (h)	6.6±1.8	6.3±2.4	5.2±1.4
RIN	6.5±0.6	6.3±1.1	6.9±1.1

Table S1. Tissue samples information. M: Male. F: Female. PMD: Post mortem delay. RIN: RNA integrity number. All data are shown as mean ± SD.

miRNA	Validated targets
hsa-miR-92a-3p	CAMKV, CASKIN1, CDK5R1, CDK5R1, GABARAPL2, GSK3B, MYO6, NRXN3, NSF, PPP1CC, PPP2R1A, SYNJ1, SYNJ1, UCHL1
hsa-miR-181c-5p	CAMK2D, GRM5, MAP2K1, NPTXR, GABRA1, GRM1, NRXN1, STXBP5
hsa-miR-210-3p	NPTX1, CAMKV, KCNAB2, NRXN1, STXBP5, NPTXR

Table S2. Validated miRNA-target interactions for candidate miRNAs
based on miRWalk2.0 database. Only selected synaptic-related targets are shown.

Patients	First diagnosis	Date	Last diagnosis	Date
P1	MCI	2006	AD	2017
P2	MCI	2007	AD	2011
P3	MCI	2006	AD	2017
P4	MCI	2006	NCI	2007
P5	MCI	2005	AD	2008
P6	MCI	2007	NFU	
P7	MCI	2006	FTD	2011
P8	MCI	2006	NCI	2010
P9	MCI	2006	AD	2017
P10	MCI	2005	NFU	
P11	MCI	2007	VD	2014
P12	MCI	2006	NFU	
P13	MCI	2007	AD	2017
P14	MCI	2006	MCI	2017
P15	MCI	2006	AD	2013
P16	MCI	2006	NFU	
P17	MCI	2005	NFU	
P18	MCI	2005	MCI	2008
P19	MCI	2005	AD	2017
P20	MCI	2005	NFU	
P21	MCI	2005	AD	2006
P22	MCI	2006	NCI	2007
P23	MCI	2005	NFU	
P24	MCI	2006	AD	2017
P25	MCI	2006	AD	2007
P26	MCI	2006	AD	2006

Table S3. Follow-up of MCI patients. P: patient. MCI: Mild cognitive impairment. AD: Alzheimer´s Disease. FTD: Frontotemporal dementia. VD: Vascular dementia. NCI: No cognitive impairment. NFU: No follow-up available.
